# Supplementary material for: Comparing the effect of cross-group friendship on generalized trust to its effect on prejudice: The mediating role of threat perceptions and negative affect
Source: PLoS One. 2021 Feb 5;16(2):e0245983. doi: 10.1371/journal.pone.0245983 (PMC7864407; doi:10.1371/journal.pone.0245983)
Supplement: Attachment — Submitted filename: Response to Reviewers.docx [file pone.0245983.s002.docx]

***Response to Reviewers***

***Reviewer #1:***

*This study investigates a topic of great importance for both the literature in social psychology and political and social interventions to increase ethnic integration. A topic that is especially relevant today as societies are growing in diversity. The topic, however, has been extensively researched before, which makes contributing to the field a bit hard. However, the authors were able to see a gap in the literature and tried to address it. That said, I think the paper has a few major shortfalls that should be addressed before publication.*

Thank you for your positive evaluation of the importance of our topic and the recognition of the contribution of our manuscript. We have taken your constructive suggestions for further improvements to heart, and set out below how we have addressed your comments.

*1. Conclusions are not supported by the data: In the hypotheses you want to test different “effect sizes”, however, the statistical analyses do not focus on this issue, but rather on coefficient values (betas) and their p-value. P-values are not a measurement of the effect size and the beta coefficients can only be a measure when certain conditions are met. Furthermore, the authors claim to demonstrate that the effect can be negligible. I would suggest the authors to either delete the references to effect size in the hypotheses or present analyses on the effect sized and the nature of the null effect. The authors could perhaps run a equivalence test for the null effect. If I am getting this wrong, I am, of course open to read the author’s counter-argument. As you will see in my specific comments, I think the authors should tone down the implications of the findings, and discuss more in-depth the limitations of the analyses they perform. Are there any limitations in their data analysis that may explain the lack of link between cross-friendship and generalized trust?*

We thank the reviewer for pushing us to clarify our approach and agree that coefficients and p-values are not sufficient to claim that our effects are large enough or practically important/significant. Indeed, trivially small effects can be statistically significant with large sample sizes. Although significance does carry information, evaluating mediation should not solely be defined in terms of statistical significance. We also agree that equivalence tests can be employed to assess whether an effect is small enough to be negligible. However, the information on which such confidence intervals are based has to be derived from external sources (such as arbitrary rules of thumb, census information, prior research, or when in conjunction with power analyses, derived from a feasible sample size; (Lakens, Scheel, and Isager 2018)). In our case, there is unfortunately very little prior research on the total effect of cross-group friendship on trust, and no prior study engaged in a mediation analysis. Therefore, we decided to assess our findings against the benchmark of an established path in social psychological literature, namely that between cross-group friendship (x), negative affect and threat (z), and prejudice (y). In practice, this means that we evaluate our effect size(s) in terms of a specified value, which is i) theoretically based on prior research in intergroup relations, and ii) simultaneously estimated for the same respondent using the same data source. Note that this approach follows the definition of effect size by Preacher and Kelly (2011: 95, our italics) as: “any measure that reflects a quantity of interest, either in an absolute sense or *as compared with some specified value*”. “Standardized effect sizes are on a meaningful scale in units of standard deviations”. McKinnon (2008: 81-85) similarly recommends evaluating standardized effect sizes of individual and mediated paths. Moreover, it is important to note that Betas derived from Structural Equation Modeling are often deemed superior since these are simultaneously estimated with multiple variables and assessed in light of measurement error (a latent variable approach) (Hancock 2001). We do discuss magnitudes in relation to each other since this is imperative to the goal of the paper, but have followed your recommendations and in the literature (Preacher and Kelley 2011), which suggest avoiding providing excessive commentary about the sizes and removed the word “negligible” in this regard throughout the manuscript.

Nevertheless, we agree that we should formally test whether our Betas statistically differ rather than eyeballing their magnitude. There are several tests available for the equality of effect sizes, such as the Wald confidence interval (CI) through Sobel’s method, likelihood-based CI, and variations of Bootstrap CI (Cheung 2007). The Bootstrap CI outperforms all since it does not assume that the data are multivariate normal, is not sensitive to sample size, and it is not symmetric around the estimated mediating effect. The latter is especially important since “the sampling distribution of a mediation effect is complicated because the mediation effect is quantified by a product of at least two parameters” (Ryu and Cheong 2017: 1). More specifically, “the sampling distribution of the product term *ab* is not normal … even though *a* and *b* are normally distributed” (Cheung 2007: 231; MacKinnon 2008: 95). That is to say, even if two effects are statistically significant their joint test is not necessarily so. For these reasons comparing Bootstrap CI and whether these would not overlap is superior to other tests. We have conducted such tests and reported these CIs, and discuss whether these do not overlap for our effect sizes of interest in the text. The results are in line with what we had reported before, which is due to the observed large differences.

“In addition, we report Bias corrected Bootstrapped Confidence Intervals (2000 iterations) for our total, total indirect, and indirect effects in order to compare the effect sizes. The Bootstrapped Confidence Intervals (BC CI) outperforms other tests since it does not assume that the data are multivariate normal, is not sensitive to sample size, and it is not symmetric around the estimated mediating effect. The latter is important because even if two effects are statistically significant their joint test is not necessarily so [57 p. 231,58 p. 95,59 p. 1].” (p. 14)

“The model also indicates a statistically significant total effect of cross-group friendship on generalized trust. Yet, this is substantially weaker than the negative total effect of cross-group friendship on prejudice. The 99% BC CI of its standardized value lies between 0.013 and 0.106. For the total effect of cross-group friendship on prejudice the 99% BC CI lies between -0.363 and -0.280. Since the highest absolute value of the path between cross-group friendship to trust and the lowest absolute value of the path from cross-group friendship to prejudice do not overlap, we can be confident that these estimated values are substantially and statistically different.” (p. 17)

“the indirect effect of friendship on trust through threat is much weaker (0.060) than that on prejudice (-0.111). If we evaluate the absolute values at the lower and upper bounds of the 99% BC CIs of these effect sizes, we see that these do not overlap (0.040 – 0.080 versus -0.137 – -0.085). We can thus be confident that these effects statistically and significantly differ in size.” (pp. 19-20)

In the previous version two of the control variables had been mistakenly omitted in parts of the model. The inclusion of these variables introduced very minor changes in the model fits and coefficients. In addition, in the most recent version of Mplus 8, instead of WRMR, SRMR is reported as one of the Goodness of Fit indicators, which we now have followed.

*2. The paper is written in a way that it is somewhat difficult to understand in some parts. The authors use complicated rhetoric and repeat information, often superfluous, a lot. I am not a native speaker myself so I am not talking about the quality of the language per se (which is fine in my opinion), but the clarity of the exposition. I would suggest the authors work a bit on the literature review section and focus on the research gap they intent to address because the goal of the paper or how the authors arrive to conclusions is not always clear. The results and discussion section are ok, although the discussion is a bit too long.*

Thank you for pointing this out. During the revision process, we have carefully edited the manuscript to clarify and simplify our exposition, remove superfluous repetitions and streamline as well as shorten the discussion section. We have thereby also followed the more detailed suggestions you made regarding the separate sections of our manuscript, and are grateful to you for pushing us further in this direction.

*Introduction and literature review*

*• The authors make a very good job at explaining and describing the research process: how and what their study will test. However, it comes across as somewhat disorganized and too technical for an introduction. I was expecting to be told why I should be interested in the topic, how the findings will fit in the literature. For example, in the last paragraph in page 3, the authors first explain that they intend to conduct a confirmatory factor analysis and then they finish the paragraph explaining what we can learn from such analysis. I would suggest the authors that they reverse this order and first state what they want to learn and why, and only then explain how they plan to do it.*

*• Page 4: “Sufficient studies confirm…” Sufficient is something to be judged by the audience so I would recommend the authors to write something that refers just to the amount such as a lot, few, many studies…*

*• Page 5, line 98: “stimulating positive subjective orientations towards”. This is maybe a very well formulated description for a psychology audience but PlosOne is a journal targeting a general audience so I would recommend the authors to reduce the use of jargon. It makes the paper quite difficult to follow.*

*• Page 5: the authors discuss that cross-group friendships meet all Allport’s conditions to reduce prejudice. I think this fact should be introduced earlier.*

We have followed each of these suggestions to simplify and clarify our introduction.

*Hypotheses*

*• Can the authors elaborate on why they compare the 2 causal effects sizes? Why is the comparison of the size effect interesting? What would imply a disparity of the effect sizes? If the direct effect of cross-friendship in trust has not been yet tested as the authors claim? Shouldn’t they test this first?*

Our primary interest lies in the total, direct, and indirect effects of cross-group friendship on trust. Yet, as mentioned in relation to your first comment, we use the results on prejudice as a benchmark to assess our estimated effect sizes. The comparison between the observed effects on trust and prejudice thus is important to present and discuss since it allows us to embed our findings more closely in the existing literature (which studied prejudice, but not yet trust).

*Results*

*• In page 17, the authors conclude that “The model also indicates a weak and statistically significant total effect of cross-group friendship on generalized trust and a relatively substantial negative total effect of cross-group friendship on prejudice. Therefore, we can refute H1A (an equally strong total effect), but not H1B (comparatively weaker total effect of cross-group friendship on generalized trust). Both these paths will next be examined including the mediators in the models.” How do you measure effect size? Does you analytical strategy actually test these hypotheses?*

Mediation can be defined in terms of the following equation c = c’ + a*b (in this example there is only one y-variable), whereby c stands for the total effect, c’ for the direct effect, and a*b for the indirect effect. Our hypotheses H1A and H1B test for the equality of the magnitude of c (the total effects). Our hypotheses H2A and H2B test for the equality of the magnitude of the indirect effects. Note that in our case there are multiple y-variables and mediators present, the effects of which are added.

Effect size was interpreted in terms of the estimated standardized coefficients, although we agree with your earlier comment that there are important assumptions underlying the validity of this approach. That being said, an important insight can be obtained from observing in our analysis that cross-group friendship is associated with lower levels of prejudice in a substantial and statistically significant manner (as also shown in previous work), while it only has a small effect on generalized trust (a new finding from our analysis).

*• I do not understand the distinctions made by the authors between direct and indirect effects. Wouldn’t all effects be direct until the mediators are introduced in the model? My knowledge of mediation analysis in very limited so I would appreciate if the authors could elaborate in this topic. The explanation doesn’t need to go in the paper.*

It is true that in a model without mediators the total effect of variable X on variable Y and the direct effect of variable X on variable Y will be the same. In such a setting, there thus is perfect equivalence between the ‘total’ and ‘direct’ effects, and we then would abstain from adding the word ‘total’ or ‘direct’. Yet, once mediators are introduced, this is no longer true. Then it becomes possible to separate the total effect of variable X on variable Y into a direct effect of variable X on variable Y and an indirect effect of variable X on variable Y via variable Z. The total effect then is defined as the sum of all direct and indirect effects linking the variables X and Y (Sobel 1987).

*Conclusion*

*• Page 21 lines 400-401, the authors state “our findings demonstrate that the total effect of cross-group friendship on generalized trust is statistically significant, but rather weak.” I believe the authors overstate the meaning of their findings. First, I would like the authors to tone down a bit the statements, 2) the authors should distinguish between “we didn’t find an effect” and “there is no effect”, 3) explain what could have failed (is the operationalization of the concept maybe affecting the results in any way?), and 4) perhaps conducting equivalence testing on the effect would be a nice idea? That way, the authors could argue that the effect is not larger than the minimum effect of interest. This is just a suggestion. The authors can tell me whether they think this is a good idea.*

We agree that there is an important distinction between “we didn’t find an effect” and “there is no effect”, and we have been more precise in our wording in the revised manuscript. We thereby also toned down the language. It is indeed perfectly possible that we failed to pick up stronger effects due to the operationalization of our concepts. Still, we should note in this respect that we have followed standard operationalizations for both prejudice and trust, such that our findings are based on the state-of-the-art in previous work. We have been more explicit about this in the revised version of the manuscript.

“Using a number of structural equation models, we observe that the total effect of cross-group friendship on generalized trust is statistically significant, but rather weak. There are also statistically significant indirect effects of cross-group friendship on generalized trust, which run primarily via perceptions of threat. The mediating role of negative affect could not be supported in this context. Overall, cross-group friendship thus appears to relate to lower levels of perceptions of threat, and such perceptions of threat are associated with lower levels of generalized trust.” (p. 20)

“Finally, it is possible that our (weak) findings are affected by the operationalization of our key concepts. While a common concerns for all empirical research into social and psychological concepts, we should note that we follow standard operationalizations for both prejudice and trust. Even though these operationalizations may be imperfect, this implies that we are capturing the same ‘prejudice’ and ‘trust’ (whether or not combined with other things) as in previous scholarship. Still, robustness check using alternative operationalizations would be important to substantiate our findings.” (p. 22)

*• In the conclusion, the authors devote an extensive part to the limitations. While I do think that the limitations should be highlighted, it’s be better if the authors could say how the limitations affected the findings and how or whether these limitations may be overcome. Particularly, I have trouble with the paragraph on the direction of causality as this is contradicting the section of the direction of causality. Should I then trust previous literature in this or not? What efforts have you made, if any, to overcome this general limitation?*

Thank you for pointing this out. We have rephrased our limitations section to indicate how these limitations might affect the results presented in the manuscript. We have also revised our paragraph on the direction of causality in the conclusion to make it consistent with the discussion of this same issue earlier in the manuscript. In our view, previous work on the implied direction of causality can be trusted, but overcoming this limitation would require longitudinal data with exogenous variation in cross-groups friendships (i.e. unrelated to self-selection). A few recent studies based on experimental research designs have taken steps in this direction (Finseraas and Kotsadam, 2017; Finseraas et al., 2019), but such data was unfortunately unavailable to us. We have discussed this in the revised version of our concluding discussion.

*• Line 456: The authors again use the work “demonstrate” and I would suggest hem to tone down this a little bit or perform analyses that can support this asseveration.*

*• Last paragraph: “As we demonstrate here, the relationship between cross-group friendship and generalized trust is less strong than that between cross-group friendship and prejudice. When studying generalized trust at the individual level, we therefore suggest analysts should consider alternative mechanisms…” Again, demonstrate is too strong. However, this implication is in fact the most interesting.*

We have toned down the language and avoid the use of the word ‘demonstrate’ (or equivalents).

*• Are the scripts for data analyses available?*

Yes. All scripts will be made available.

***Reviewer #2:***

*This is an interesting paper that studies an important and under-analyzed topic, the role of cross-group friendship on trust and prejudice. In general terms, I like it, although I think that the theory is under-developed and the results are probably driven to a certain extent by the indicators of prejudice and trust used in the paper.*

Thank you for your positive evaluation of our manuscript, and your constructive suggestions for further improvements. We set out below how we have addressed your comments.

*- The idea is that cross-group friendship may enhance a broader willingness to trust in others. But why trust in general others? Cross-group friendship could have an effect on trust in the ethnic group of your friend, so, perhaps, we should see an effect of cross-group friendship on out-group trust, but not so much on social trust. Friendship clearly positively affects your beliefs about your friend’s trustworthiness (actually, friendship and trust are closely related, as Cicero would say). It is not so clear to what extent it affects your beliefs about general others. Clearly, your friends are not a representative sample of society, so in principle, friendship would affect just particularized forms of trust. Moreover, if the cross-group friendship is towards a member of a minority ethnic group, even if the truster considers that her friend is somewhat representative of her ethnic group, this may not have much force in his general assessment of the distribution of types in the wider society. What I mean is that his assessment of the distribution of types in society could be to a big extent disconnected of his beliefs about the trustworthiness of a specific ethnic group.*

We agree that one would first of all expect to see an effect of cross-group friendship on out-group trust. However, our argument is that such effects – once established – might then spill over to broader-based social trust via so-called secondary transfers. We have been more detailed about the underlying theoretical argument in the revised version of the manuscript. Clearly, however, whether or not such hypothesized effect on social trust materializes remains an empirical question, which we address in our analysis.

“Generalized trust not only requires reduced prejudice or lower levels of negative emotions towards a specific out-group. It also implies extending this towards unknown people. Some argue that intergroup theory is applicable to the analysis of generalized trust due to ‘secondary transfer effects’. These occur when positive attitudes towards encountered individuals are generalized to a wider group [15,28]. If so, cross-group friendship may enhance a broader willingness to trust [29].” (p. 5)

*- I have some problems disentangling trust from prejudice. In what sense these beliefs can be differentiated from the belief about someone’s trustworthiness? Some of the reasons for not being willing to send children to a school with a majority of immigrants, or to move to a district with many immigrants may be based on beliefs neither related to prejudice or to trust. For example, you may be reluctant to send your children to schools with a majority of immigrants because you have seen (in official reports, perhaps) that the quality of the education in these schools is relatively low, or you may not want to move to a district with many immigrants because you fear that this would affect to the future price of your home (perhaps not because of your prejudice but because of other people's prejudice against immigrants). Likewise, you can be in favour of giving the employer the right to only employ nationals because, as a national, it benefits you. But to the extent that these indicators do capture prejudices, in what sense do they differ from trust? It is remarkable that there is no definition of what prejudice is in the paper (neither of what trust is, for that matter). But if prejudice is the notion that someone is dishonest, or do not play by the rules of the host country, how is this different from the belief on someone’s trustworthiness? I wonder whether the results can be partly explained because what the authors are measuring is not actually prejudice, but something that combines prejudice (which, as I said, is a belief that should be very close to trusting) and other things.*

These are very valid points, and we very much agree that there may well be other elements unrelated to prejudice or trust that can explain why people would be unwilling to send their kids to certain schools. Yet, the activities mentioned in the survey data we use are commonly thought to be driven in part by feelings of prejudice, and we have taken this standard operationalization to allow for comparability with previous findings. That being said, we agree that the concepts of trust and prejudice are closely related on a conceptual level, which makes it particularly important clearly to separate them in empirical work. This is the main reason why our analysis started out by testing whether our data captures two distinct concepts. We have now also provided definitions of prejudice and trust in our revised manuscript (and apologize for their absence in the previous version).

“In a first step, we assess to what extent generalized trust, threat perceptions, negative affect, and prejudice are separate latent constructs as well as how strongly these constructs are related. This is done using a Confirmatory Factor Analysis (CFA) framework, and is essential for the evaluation of the measurement part of our structural models that follow.” (p.4)

“There are important conceptual differences between generalized trust (trust towards people we do not know) [12], particularized trust (trust towards people we know) [13], and out-group trust. The latter is trust in ethnic groups one believes not to share a common descent with [2]. Generalized trust is the belief that unknown people do not do us harm [16], which differs from prejudice. Prejudice is “an antipathy based upon a faulty and inflexible generalization”[11 p. 9]. It involves the rejection of and opposition to contact with the out-group. Pettigrew and Meertens [17 p. 58] add to this definition that “prejudiced attitudes tend to form ideological clusters of beliefs that justify discrimination”.” (pp. 2-3)

Finally, it is possible that our findings reflect the operationalization of our concepts. This is by definition true for all empirical research into social and psychological concepts. Still, we should note that we follow standard operationalizations for both prejudice and trust. This implies that we are capturing the same ‘prejudice’ (whether or not combined with other things) as previous scholarship. Nonetheless, these operationalizations may be imperfect, and following your suggestion we have been more explicit about this in the revised version of the manuscript.

“Finally, it is possible that our (weak) findings are affected by the operationalization of our key concepts. While a common concerns for all empirical research into social and psychological concepts, we should note that we follow standard operationalizations for both prejudice and trust. Even though these operationalizations may be imperfect, this implies that we are capturing the same ‘prejudice’ and ‘trust’ (whether or not combined with other things) as in previous scholarship. Still, robustness check using alternative operationalizations would be important to substantiate our findings.” (p. 22)

*- The authors dismiss the problem of endogeneity in a rather cavalier manner; it may be true that if the results refute the hypothesis, there is no point in conducting research with a stronger analysis; but they have to be conscious that with cross-sectional data and not causality test (such as an instrumental variable approach, for example), the results just say that it could exist a causal relationship going from cross-group friendship to prejudice, but it could also be the other way round: less prejudiced people are more likely to have more friends from other ethnic groups.*

We did not mean to dismiss the problem of endogeneity, and agree that the relation between friendship and trust/prejudice may also run in the opposite direction. You are right that our data and research design cannot conclusively rule out the presence of important effects of cross-group friendship on trust. We have therefore been more careful in our wording about the implications for (not) conducting further research in this direction, as well as about the issue of causality more generally. We have added two citations that take an experimental approach for illustration (Heining Finseraas and Kotsadam 2017; Henning Finseraas et al. 2019).

“That being said, our cross-sectional analysis cannot establish causality. Although one might pose that investigating the relations under analysis in a cross-sectional survey is futile, we believe our analysis nonetheless retains significant merit by assessing and highlighting the potential role of mediators in the contact-trust relation at the individual level.” (p.8)

“Despite these contributions, our study has also some limitations. One limitation is that the direction of the causal chain cannot be established with certainty in our dataset. While this causality issue can be overcome via panel data, the small effect sizes observed in our analysis suggest that we may need frequent measurements over a lengthy period to decompose time varying (macro and micro), and time invariant (i.e. individual-level fixed) effects. Such a panel will be a costly endeavour with potentially unsubstantial effect sizes suggested by our results. As an alternative approach, one could consider experimental research designs, which allow for much stronger causal inferences. A few recent studies have taken this route to study the relation between personal contact and anti‐immigrant sentiments as well as trust [65,66].” (p. 21).

*- A minor point, the authors claim that cross-group friendship meets the condition of equal social status: Is this the case when people have a different ethnic background? Has a member of the minority group the same social status than the member of the majority?*

Status is unlikely ever to be fully equal even in cross-group friendships. Yet, friendship implies that status differentials between the involved individuals are likely to be small enough (or, at least, perceived to be small enough), certainly in comparison to mere contact between individuals (where status differences may remain – and be perceived as – much more pronounced). Following your comment, we have rephrased ourselves and no longer state that cross-group friendship meets the condition of equal social status (which was too strong). Instead, we argue that friendship mitigates (perceived) status differentials and thereby goes towards meeting the condition of equal (or, at least, similar) perceived social status.

“Yet, cross-group friendship meet key requirements specified by Allport [11] as crucial conditions for the contact hypothesis to work: i.e. friendship mitigates (perceived) social status differentials between the involved individuals (…).” (p. 2)

**REFERENCES**

Cheung, Mike W.L. 2007. “Comparison of Approaches to Constructing Confidence Intervals for Mediating Effects Using Structural Equation Models.” *Structural Equation Modeling: A Multidisciplinary Journal* 14(2): 227–46.

Finseraas, Heining, and Andreas Kotsadam. 2017. “Does Personal Contact with Ethnic Minorities Affect Anti-Immigrant Sentiments? Evidence from a Field Experiment.” *European Journal of Political Research* 56(3): 703–22.

Finseraas, Henning et al. 2019. “Trust, Ethnic Diversity, and Personal Contact: A Field Experiment.” *Journal of Public Economics* 173: 72–84.

Hancock, Gregory R. 2001. “Effect Size, Power, and Sample Size Determination for Structured Means Modeling and MIMIC Approaches to between-Groups Hypothesis Testing of Means on a Single Latent Construct.” *Psychometrika* 66(3): 373–88.

Lakens, Daniël, Anne M. Scheel, and Peder M. Isager. 2018. “Equivalence Testing for Psychological Research: A Tutorial.” *Advances in Methods and Practices in Psychological Science* 1(2): 259–69.

MacKinnon, David. 2008. Introduction to Statistical Mediation Analysis *Introduction to Statistical Mediation Analysis*. New York, NY.: Lawrence Erlbaum Associates.

Preacher, Kristopher J., and Ken Kelley. 2011. “Effect Size Measures for Mediation Models: Quantitative Strategies for Communicating Indirect Effects.” *Psychological Methods* 16(2): 93–115.

Ryu, Ehri, and Jeewon Cheong. 2017. “Comparing Indirect Effects in Different Groups in Single-Group and Multi-Group Structural Equation Models.” *Frontiers in Psychology* 8: 747.

Sobel, Micheal E. 1987. “Direct and Indirect Effects in Linear Structural Equation Models.” *Sociological Methods & Research* 16(1): 155–76.
